# Measuring Thickness-Dependent Relative Light Yield and Detection Efficiency of Scintillator Screens

**DOI:** 10.3390/jimaging6070056

**Published:** 2020-06-29

**Authors:** William C. Chuirazzi, Aaron E. Craft

**Affiliations:** Idaho National Laboratory, Idaho Falls, ID 83415, USA; aaron.craft@inl.gov

**Keywords:** neutron imaging, neutron radiography, digital imaging, scintillator screen, scintillator development, scintillator performance, detection efficiency

## Abstract

Digital camera-based neutron imaging systems consisting of a neutron scintillator screen optically coupled to a digital camera are the most common digital neutron imaging system used in the neutron imaging community and are available at any state-of-the-art imaging facility world-wide. Neutron scintillator screens are the integral component of these imaging system that directly interacts with the neutron beam and dictates the neutron capture efficiency and image quality limitations of the imaging system. This work describes a novel approach for testing neutron scintillators that provides a simple and efficient way to measure relative light yield and detection efficiency over a range of scintillator thicknesses using a single scintillator screen and only a few radiographs. Additionally, two methods for correlating the screen thickness to the measured data were implemented and compared. An example ^6^LiF:ZnS scintillator screen with nominal thicknesses ranging from 0–300 μm was used to demonstrate this approach. The multi-thickness screen and image and data processing methods are not exclusive to neutron scintillator screens but could be applied to X-ray imaging as well. This approach has the potential to benefit the entire radiographic imaging community by offering an efficient path forward for manufacturers to develop higher-performance scintillators and for imaging facilities and service providers to determine the optimal screen parameters for their particular beam and imaging system.

## 1. Introduction

Radiography measures the neutron transmission through a sample to non-destructively visualize its internal condition. Neutron radiography has been utilized in a multitude of applications including those of nuclear fuels [1,2,3,4,5], cultural heritage objects [6,7,8], and industrial applications such as fuel cells [9,10,11,12,13] and turbine blade analysis [14,15,16].

Digital neutron imaging systems are an essential component of state-of-the-art neutron imaging facilities. The most common type of digital neutron imaging system uses a digital camera that is optically coupled to a scintillator screen. A neutron scintillator screen absorbs neutrons and produces photons, which are then recorded by a digital camera to produce a digital image. Neutron scintillator screens consist of common scintillator materials like those used for X-rays but include a neutron absorber to make them sensitive to neutrons. 

In addition to being a limiting factor of an imaging system’s spatial resolution, the neutron scintillator screen also determines the neutron capture efficiency (i.e., detection efficiency) of the imaging system [17]. High detection efficiency of the scintillator screen is especially crucial because of the limited availability of neutron beamtime and high costs associated with nuclear reactor and accelerator operations. Additionally, as long as the photon counting statistics sufficiently represent the number of neutrons captured, the image quality is driven by the neutron counting statistics of the scintillator screen as determined by the detection efficiency.

Detection efficiency is the percentage of neutrons absorbed by the scintillator screen. Scintillator screens with higher detection efficiency provide superior imaging statistics when compared to an exposure of the same time taken with a scintillator with lower detection efficiency. Detection efficiency is a critical criterion of a scintillator’s performance and has been characterized for many other scintillator screens [18,19]. A common method to measure detection efficiency uses a photomultiplier tube and multichannel analyzer to perform pulse height analysis on the scintillator screen’s signal, which is then compared to the signal of a reference detector [20,21]. Not only does this require expensive equipment, but it also requires additional measurements and is thus more time consuming. Using a separate detector to compute neutron time of flight can also be used to determine detection efficiency, but this again necessitates additional equipment and measurements [22]. This work offers a relatively simple method of measuring the neutron detection efficiency of a scintillator screen that does not require extra equipment or complicated techniques. 

Developments in neutron scintillators seek to increase their image quality while decreasing the amount of beamtime necessary to acquire an image. Scintillators with improved neutron detection efficiency allow for the quicker acquisition of radiographs. This reduces the time required to acquire a full set of neutron radiographs suitable for neutron tomography [23,24]. Additionally, high-speed tomography applications can realize improved image quality without increasing acquisition time [25]. Neutron scintillators with improved detection efficiency can reduce costs and increase throughput because less beamtime is needed for each radiograph.

Unfortunately, development of improved neutron scintillator screens has historically required significant time and cost to fabricate and test new screens. Improved testing and evaluation methods to more efficiently evaluate screen performance would benefit manufacturers of scintillator screens seeking to produce ever-better screens and those who operate neutron imaging facilities seeking to determine which screen parameters provide the desired performance with their particular neutron beam and imaging system. 

This work offers a new way to test and evaluate scintillator screens by implementing a variable-thickness scintillator screen geometry with a new efficient test method that simultaneously provides relative light output and detection efficiency for a continuous range of scintillator screen thicknesses.

## 2. Variable-Thickness Scintillator Screen Approach

Traditionally, new neutron scintillator screens have been designed using a scintillator layer of a single thickness [26,27,28]. Single thickness scintillators have been used because scintillator screens of a discrete thickness are needed for neutron radiography and tomography to achieve uniform illumination of the field of view. Using single thickness scintillator screens for testing multiple screens can be time consuming and expensive. Measurements taken with a single thickness screen yield only one data point, and many data points are required to understand screen performance over a range of thicknesses.

Rather than fabricate multiple sensors of discrete scintillator layer thicknesses, a range of thicknesses was simultaneously tested by using a variable-thickness screen. This approach uses a scintillator layer that continuously varies in thickness across the entire scintillator screen, creating a wedge shape. The wedged screens were created by first mixing a homogeneous slurry of ^6^LiF:ZnS, then depositing a 300 μm thick layer onto the substrate. A rigid razor blade was used to scrape the scintillator layer away until the target maximum and minimum thicknesses were reached with a monotonic gradient in between them. A jig was used to keep the blade at the desired angle and thickness. 

**Figure 1:**
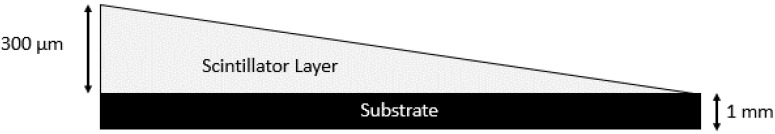
Schematic diagram of a wedged scintillator (*not to scale*). The wedge allowed a range of thicknesses to be simultaneously tested.

Figure 1 shows a diagram of the side view of a wedged scintillator with the wedge shape exaggerated for emphasis. For the screen used to demonstrate the method in this work, a wedged screen was fabricated with a maximum target thickness of 300 μm that decreased to a thin layer measuring just a few microns thick, deposited on a 1 mm thick aluminum substrate. Maximum scintillator screen thickness was measured with two different coating thickness gauges, both of which used an eddy current probe to determine the scintillator layer thickness. The nominally 300 μm thickness region was found to have a thickness of 326 μm.

Neutron sensor designs that separate the converter and scintillator layers can utilize a double-wedged structure. In this approach the separate converter and scintillator layers are both wedged, but the converter wedge and scintillator wedge are placed perpendicularly to each other so that thickness is varying in one layer but remains constant in the other. The double-wedge structure allows a multitude of converter and scintillator thickness combinations to be tested simultaneously. This radically expedites the testing of new scintillator screens, because hundreds of thickness combinations can be tested with a single screen. Previous methods would require a separate screen for each thickness combination. 

**Figure 2:**
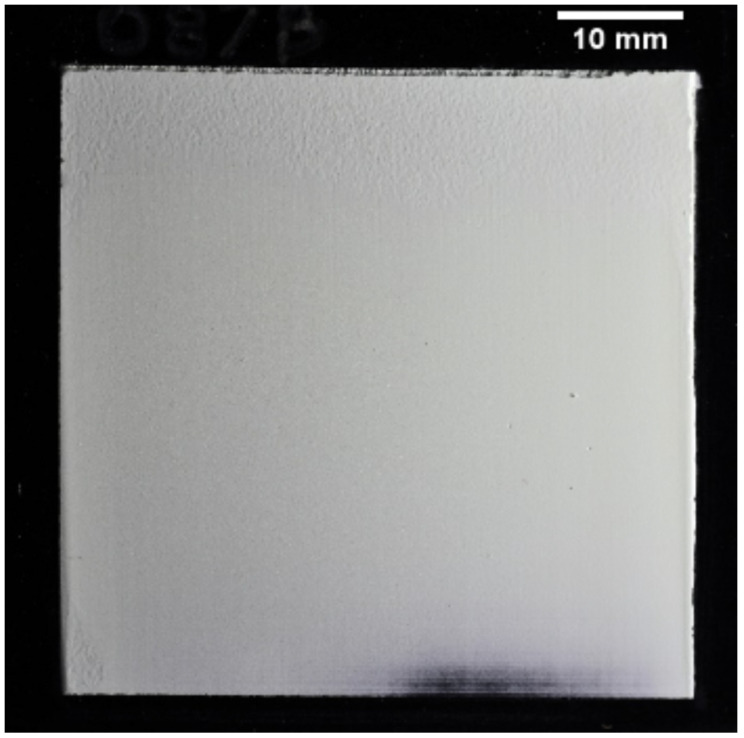
Wedged ^6^LiF:ZnS scintillator photograph with oblique lighting to emphasize surface texture. Measured scintillator thickness at the top of the screen is 326 μm and decreases to nominally 0 μm at the bottom of the screen.

A wedged ^6^LiF:ZnS screen with a target maximum thickness of 300 μm was fabricated to demonstrate this concept and determine the optimum scintillator thickness. The ^6^LiF:ZnS had a 2:1 mix ratio by weight. The median size of the scintillator particle sizes was 6.7 μm, and the scintillator was deposited on a 1 mm thick aluminum substrate with a black satin finish to reduce light backscatter from the scintillator. An optical photograph of the scintillator, shown under oblique lighting to emphasize surface features, is shown in Figure 2.

## 3. Neutron Scintillator Light Output and Detection Efficiency

Scintillator screen light output is a common characteristic used to describe scintillator screen performance. Light yield, a measure of photons produced under neutron exposure, is one of the most commonly measured scintillator characteristics. This is because sufficient light yield is necessary to produce distinguishable contrast in a neutron radiograph. A scintillator that does not produce enough light may be incapable of producing a useful neutron radiograph.

It is true that the camera must receive sufficient light from the scintillator to produce an image. However, as long as the light collection sufficiently represents the number of neutrons absorbed by the screen, the neutron image quality is primarily driven by the number of neutrons passing through the object and detected by the screen. As neutrons interact with the imaging object, they carry attenuation and geometric information about the sample to the scintillator screen where the image information originates [29]. Therefore, the neutron detection efficiency of a scintillator screen is a key evaluation parameter when quantifying the performance of new scintillator screens. 

Despite the importance of detection efficiency in image quality, a neutron scintillator must produce sufficient photons to provide adequate signal to the camera to represent the neutrons absorbed by the screen. A general criterion is that a neutron scintillator screen should ideally fill 80% of a digital camera’s dynamic range, but this may vary depending on exposure time to produce a useful radiograph and the available beamtime. The exposure time should be reasonably short to allow for the practical application of radiography and tomography.

## 4. Procedure for Scintillator Screen Characterization Measurements

The method developed in this work requires acquisition of only a few radiographs to determine three valuable performance characteristics of scintillator performance: light output, detection efficiency, and qualitative number of neutrons per gray level. Light output is measured by a radiograph directly of the test-screen. A flat-field radiograph is then acquired of some standard single-thickness scintillator screen (e.g., a ^6^LiF:ZnS screen). Detection efficiency is measured by placing the test-screen on the standard screen to acquire a radiograph of the test-screen, applying the flat-field correction, and processing the image. These four images (test-screen light output radiograph, dark-field image, flat-field image, test-screen attenuation radiograph) are then processed to determine relative light yield and detection efficiency. The process to acquire the four images and the post-processing procedure are described in the following sections.

This method could be used in any facility, but the measurements taken for this study of the ^6^LiF:ZnS screen in this work used to demonstrate this procedure were conducted at the ANTARES cold neutron beam located at the FRM-II reactor in Garching, Germany [30]. All radiographs were acquired using an L/D ratio of 540 which produces a neutron flux of 2.6 × 10^7^ n/cm^2^/s at the sample position. An Andor iKon L 936 camera (Oxford Instruments, Abingdon, United Kingdom) mounted in a light-tight camera box was used to obtain the neutron radiographs for this study. The iKon L 936’s camera chip contained 13.5 μm × 13.5 μm pixels in a 2048 × 2048 array and offered a 16 bit dynamic range [31]. In this work, the camera was cooled to −90 °C to reduce thermal noise. The total field-of-view was 16.1 cm × 16.1 cm, although the image was cropped to ~6.3 cm × ~6.3 cm to focus on the scintillator screen.

### 4.1. Image Acquisition Procedure

**Figure 3:**
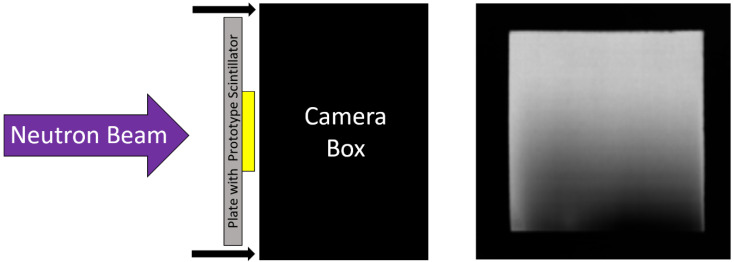
(Left) Diagram of a light yield measurement. The test-screen is optically coupled directly to the camera. (Right) A neutron radiograph acquired with a ^6^LiF:ZnS wedge-type scintillator screen. Non-linearity of the wedge is visible in the radiograph which is accounted for using the method described in this work.

The relative light yield between scintillators was measured by exposing scintillators to the same neutron fluence and comparing the grayscale values of the resulting images. The setup for light-yield measurement is depicted in Figure 3, where the test-screen is optically coupled directly to the camera.

A standard scintillator screen was mounted in the imaging system and optically coupled to the digital camera. A dark-field image was acquired with the beam off to measure the camera’s response due to the thermal electronic noise in the camera chip, and a flat-field image was acquired of the standard screen with the flat beam to account for non-uniformities in the neutron beam and standard screen. 

**Figure 4:**
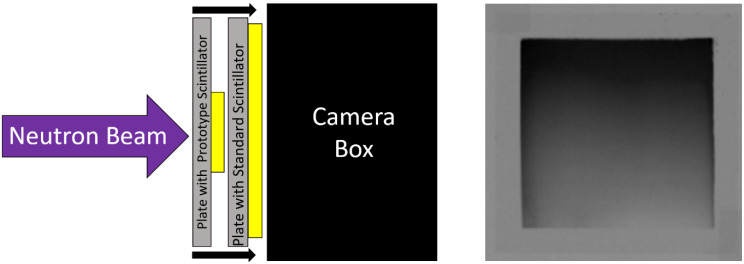
(Left) Diagram of the setup for a detection efficiency measurement. The test-screen is radiographed by a standard neutron scintillator screen that is optically coupled to the camera. (Right) A neutron radiograph taken with a commercial scintillator screen of a wedge-type ^6^LiF:ZnS scintillator screen.

The test-screen was then mounted to the imaging system on the source-side of the standard screen, as depicted in Figure 4. As the neutron beam passes through the test-screen, neutrons were attenuated, and light was produced. However, the light from the test-screen was blocked by the standard scintillator screen’s aluminum substrate and did not reach the camera. The test-screen attenuates neutrons, reducing the neutron flux experienced by the standard screen which results in a lower light output.

### 4.2. Image Post-Processing Procedure

Light yield of a neutron scintillator, and thus the grayscale value of the resulting image, *LY*(*x,y*), is proportional to the neutron flux experienced by the scintillator screen because the neutron–photon production reaction rate is directly proportional to the neutron flux. This relationship is shown in Equation (1), where *RR* is the reaction rate (photons produced), *Σ* is the macroscopic cross-section of the scintillator screen, and *f* is a conversion factor encompassing many phenomena including: escape probability of a daughter product in the converter, photons produced per neutron absorbed, photon escape probability from the scintillator screen, solid angle emission distribution from the screen towards the camera, transmission efficiency of optical components, and camera detection efficiency [32].

(1) Grayscale Value ∝RR=Σ⋅φ⋅f

The Beer–Lambert law, shown in Equation (2), describes the exponential attenuation of the neutron flux, *φ*, passing though the test-screen [30]. The initial neutron flux, *φ*_o_, undergoes exponential attenuation based on the macroscopic cross-section, *Σ*, and the thickness, *t*, of the test-screen, as shown in Equation (2). Beam hardening occurs where any poly-energetic neutron beam passes through an absorber and would require separate measurements to determine the appropriate beam hardening factor to account for the higher neutron flux present than predicted by the Beer–Lambert law without the beam hardening correction factor. Neutron build-up caused by scattering was negligible for ^6^LiF:ZnS, because the absorption cross-section was much greater than the scattering cross-section (Σ_a_ >> Σ_s_), so a buildup factor is not included in the calculation.

(2) $$\varphi \left(t\right)=\varphi_{0}∙e^{-\Sigma ∙t}$$

Equation (3) calculates the percentage of neutrons transmitted through the test-screen. Note that *Σ* and *f* cancel out in the ratio, leaving only the ratio of the attenuated and unattenuated neutron flux values.

(3) $$\%Transmission=\frac{RR_{Test\;Screen}}{RR_{Standard\;Screen}}=\frac{\Sigma ∙\varphi \left(t\right)∙f}{\Sigma ∙\varphi_{0}∙f}=\frac{\varphi \left(t\right)}{\varphi_{0}}$$

Grayscale values of a radiographic image are proportional to the screen’s reaction rate, so *φ*_0_ is directly proportional to the grayscale value of the flat-field image, *I*_0_(x,y), and *φ*(*t*) is directly proportional to the grayscale value of the radiograph of the test-screen, *I_t_*(*x,y*). Also, detection efficiency is percent-attenuation, which is unity minus percent-transmission, by definition. Therefore, Equation (3) can be reformulated in terms of grayscale values to calculate detection efficiency (*DE*) for each pixel position (*x,y*), as shown in Equation (4).

(4) $$DE\left(x,y\right)=1-\frac{I_{t}\left(x,y\right)\;}{I_{0}\;\left(x,y\right)}$$

For areas of the image outside the test-screen, *I*_0_(*x,y*) = I*_t_*(*x,y*), so *DE*(*x,y*) = 0 because there is no test-screen to attenuate the beam. For regions of (*x,y*) within the test screen, *I*_0_(*x,y*) > *I_t_*(*x,y*), leading to *DE*(*x,y*) values between 0 and 1, as expected.

This calculation is applied for the entire image area simply by dividing the radiograph of the test-screen by the flat-field image and subtracting the result from 1.

### 4.3. Correlation of Neutron Absorption to Light Yield

Another valuable parameter that can be used to evaluate a neutron scintillator screen’s performance is neutrons per graylevel. Although not an absolute measurement because of the unknown factors associated with the multi-phenomena correction factor f related to the imaging system itself, these factors are consistent among measurements using the same imaging system, which allows for a relative comparison. Neutrons per graylevel is calculated by dividing the detection efficiency of the test-screen, *DE*(*x,y*), by its light yield, *LY*(*x,y*), as shown in Equation (5).

(5) $$Neutrons\;per\;Graylevel=\frac{DE\left(x,y\right)}{LY\left(x,y\right)\;}$$

Note that for this calculation to work, the test-screen must be placed in the exact same position (*x,y*) for both the light-yield radiograph and the radiograph of the test-screen used to calculate *DE*(*x,y*). This can be accomplished using a screen mounting mechanism that allows for such repeatability. For the example measurement used here, the imaging system used identical screen-holders for both the standard screen and the test-screen such that the test-screen remained in the same position on the same screen-mount for all measurements, and the two screen-mounts were mechanically aligned (i.e., clamped together) for the radiograph of the test-screen to calculate *DE*(*x,y*).

The neutrons per graylevel measurement qualitatively describes how many neutrons are correlated to the photons that reach the camera detector. A strong correlation of neutrons to photons, meaning there are more neutrons represented by the grayscale values of the digital image, indicates improved neutron imaging statistics that allow for a more efficient use of a camera’s dynamic range. A dim scintillator may exhibit acceptable neutron image quality as long as the camera senses enough light to adequately represent the number of neutrons absorbed by the projected region of a pixel at the image plane. If a screen emits too little light, the neutrons absorbed will not be adequately represented by the graylevels of the image (i.e., the screen is so dim that the neutrons represent too few graylevels). Conversely, if a screen emits an extraordinarily large amount of light per neutron absorbed, the graylevels of the resulting image will not correlate well to the neutron counting statistics, leading to a poor-quality radiograph (i.e., the screen is so bright that the graylevels represent too few neutrons).

## 5. Correlating Measurement Results to Scintillator Thickness

Once the measurements have been taken, the next step is accounting for the wedge-shaped scintillator’s different thicknesses. Two methods of correlating the screen’s relative light yield and detection efficiency to screen thickness are presented in the following sections.

### 5.1. The Linear Thickness Assumption Method

The first method of correlating the screen performance values to screen thickness is to assume that the slope is linear between the minimum and maximum thicknesses of the scintillator layer. Each row of pixels across the scintillator screen is assumed to have the same thickness for a wedge. The median pixel value of each row can be calculated to render a plot of measured relative light yield, detection efficiency, or neutrons per grayscale over the range of thickness. 

**Figure 5:**
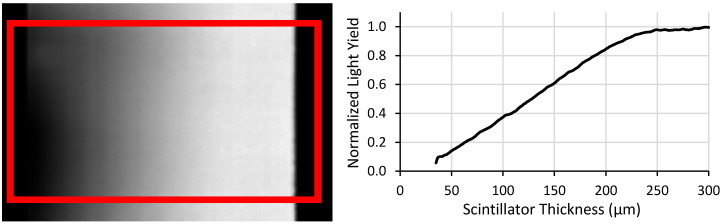
(Left) Light output image from a test-screen with the red box indicating the area that each row’s mean grayscale value was calculated. (Right) The resulting plot of light yield versus thickness.

For the ^6^LiF:ZnS screen used as an example in this work, the targeted maximum thickness was 300 µm and the minimum was assumed to be 0 µm. Although the minimum thickness in theory goes to 0 µm, the physical mean particle size of 6.7 µm prevents this. In this work, measurements were truncated below a thickness of ~30 µm to account for thicknesses representing only a few particles. The fabrication process of these screens induced some edge effects, so the edges were excluded from all analysis to prevent errors from being included in the measurement. An example of this measurement and the resulting plot is shown in Figure 5.

This approach offers a significant improvement over current methods since data for a multitude of screen thicknesses can be measured with a single measurement. The approach suggested in this work provides continuous-thickness screen performance data points, whereas multiple discrete-thickness screens would take proportionally longer to acquire that number of data points. However, this approach contains a few notable sources of error. First, this method assumes a perfect wedge shape, but the wedge is imperfect as can be seen from the non-linear light output in Figure 5. Additionally, this approach is sensitive to screen defects such as scratches on the screen’s surface. As a first order approximation, this approach can quickly and easily be implemented, but the assumptions impose some inaccuracies that are difficult to quantify.

### 5.2. Pixelwise Correlation Method

A more accurate method for correlating the screen thickness to measured data is to calculate the thickness associated with each pixel from the measured image of the scintillator screen itself. This more accurate method does not make assumptions about screen thickness based on a wedge-thickness geometry at all. In fact, a screen could be sanded or milled down to represent all thicknesses, and this method would still provide screen performance for the range of thicknesses represented by the screen.

First, the thickness the screen in each pixel of the image is calculated using the Beer–Lambert law (Equation (2)). Similar to previous calculations, *φ*_0_ is directly proportional to the grayscale value of the flat-field image, *I*_0_*(x,y)*, and *φ(t)* is directly proportional to the grayscale value of the radiograph of the test-screen, *I_t_(x,y)*. Equation (6) shows a reformulation of the Beer–Lambert law that solves for the screen thickness (*t*) multiplied by *Σf*.

(6) $$\ln\left(\frac{I_{0}\left(x,y\right)\;}{I_{t}\;\left(x,y\right)}\right)=\ln\left(\frac{\phi_{0}}{\phi \left(t\right)}\right)=\Sigma f\cdot t$$

The value of *Σf* is the same for all thicknesses. The value of *Σf* can be calculated given the image intensities at one known thickness value, then that *Σf* value can be used to calculate screen thickness for the entire screen. To perform this calculation, the images are processed according to the left side of Equation (6) using the mean pixel values in a region of known thickness. For example, the test-screen in this work had a measured maximum thickness of 326 µm (target maximum thickness was 300 μm), so the mean pixel values of the 326 μm thickness region were determined for both the flat-field and test-screen attenuation images. This value was then divided by 326 μm which gives the value for *Σf* (in units of μm^−1^). Now having the value of *Σf*, the images are processed according to Equation (7) to generate an image, *t(x,y)*, where each pixel grayscale value is the thickness in µm of the scintillator screen.

(7) $$t\left(x,y\right)=\frac{\ln\left(\frac{I_{0}\left(x,y\right)\;}{I_{t}\;\left(x,y\right)}\right)}{\Sigma f}$$

**Figure 6:**
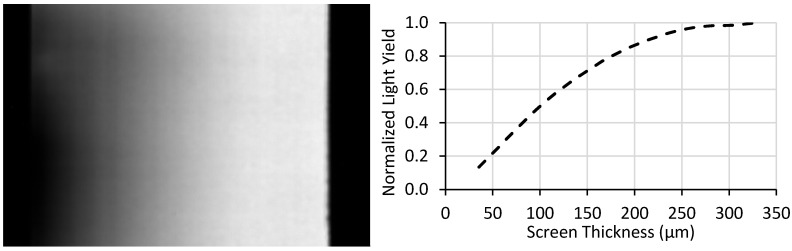
(Left) Light output image from a ^6^LiF:ZnS test-screen. (Right) The resulting plot of light yield versus thickness determined using the pixelwise correlation method.

This process can be semi-automated using an image processing software or preferred computer program. For this work, a Python script was written to perform these calculations and correlate *t(x,y)* to *DE(x,y)*, *LY(x,y)* and the ratio of the two. Once the images were correlated, the results were sorted into 1 μm bins and the median calculated for each bin to account for outliers in the data. The same light output image data used for Figure 5 was also used with the pixelwise correlation method, which yielded the analogous data shown in Figure 6.

The screen thickness can be measured directly to eliminate errors from incorrectly choosing the screen’s thickest region when initially calculating *Σf*, which was done for this calculation. Beam hardening was not accounted for in this calculation, so thickness is underestimated for thinner coatings. Light yield and detection efficiency curves would be overestimated for thinner coatings in this case. Overall, there are far fewer assumptions compared to the linear thickness method. Additionally, this pixelwise method is powerful because each data point is a valuable data point and no assumptions need to be made regarding thickness geometry at all. This approach accommodates screen defects such as scratches and thickness variations, making it a robust method. In fact, one could use high-grit sandpaper to remove material from a single-thickness screen to produce a crude multi-thickness screen, and then use this method to calculate relative light yield and detection efficiency for the range of thicknesses represented on the screen.

### 5.3. Comparison of the Two Thickness-Correlation Models

**Figure 7:**
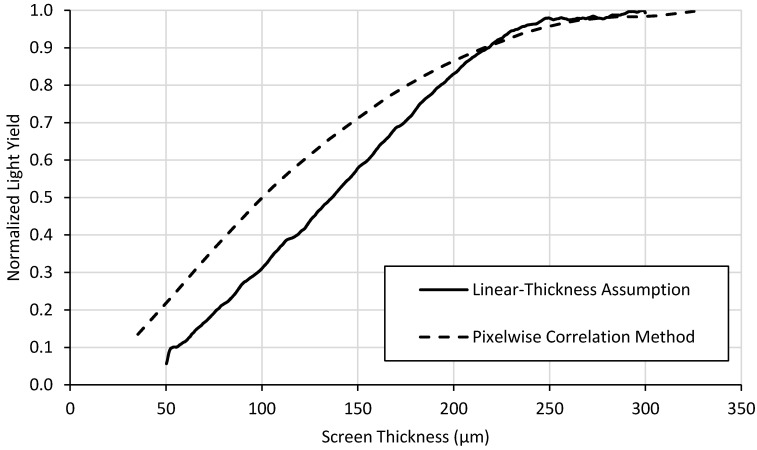
Light output as a function of thickness calculated using the two different methods for a ^6^LiF:ZnS test-screen.

**Figure 8:**
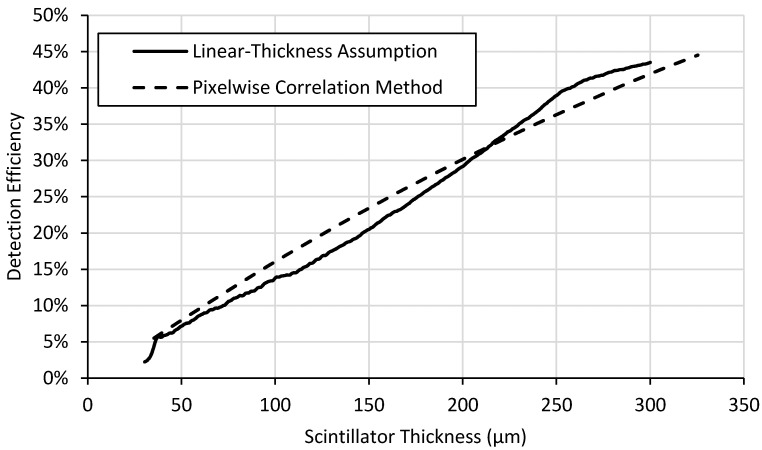
Neutron detection efficiency calculated using the two different methods for a ^6^LiF:ZnS test-screen.

**Figure 9:**
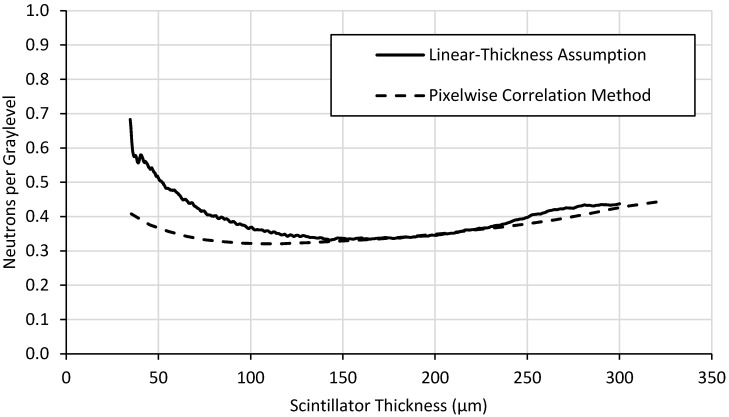
Correlation of neutron absorption to light yield using the two methods for a ^6^LiF:ZnS test-screen.

Two different methods were developed to analyze wedge-type neutron scintillator screens, the results of which are shown in Figure 7, Figure 8 and Figure 9.

The light yield plots exhibit the expected behavior for scintillator screens. Light output increases rapidly with increasing thickness for thinner screens. Self-absorption of emitted light by the screen material itself reduces the slope of the curve as thickness increases. Also, attenuation of the neutron beam with increasing thickness also reduces the slope of the curve with increasing thickness and would even lead to a negative slope for very thick screens. The detection efficiency in Figure 8 shows the expected continuous exponential attenuation with thickness. This expected behavior is exhibited in the measured data but fits the pixelwise correlation method curves far better than the curve produced by the linear-thickness assumption method. 

The correlation of neutron absorption to light yield is depicted in Figure 9. The value of this parameter is to make relative comparisons between scintillator screens to determine which screens’ light yield correlate most closely to neutron statistics. A scintillator with a higher correlation of neutron absorption to light yield is recommend when choosing between screens of similar detection efficiency and spatial resolution. 

Light output reaches plateaus around 200 µm thick (Figure 7) while detection efficiency (Figure 8) and neutron counting statistics (Figure 9) continue to increase. Thicker screens will provide lower spatial resolution, so the thinnest possible screen that provides the desired light output is preferred. Thus, a ^6^LiF:ZnS screen ~200 μm thick might represent a good balance of screen performance, i.e., light output, detection efficiency, and spatial resolution, depending on the particular application. Although detection efficiency continues to increase for thicker scintillator layers, creating screens of this composition beyond 200 μm thick will have slightly higher light output but will likely exhibit poorer spatial resolution because of internal photon scattering effects. This method does not provide information about spatial resolution, but a thinner screen would be preferred that would meet the desired spatial resolution performance. 

The light yield behavior increases with thickness, but its rate of increase diminishes with increasing thickness due to the neutron self-shielding and light opacity of the screen itself. The screen attenuates neutrons which reduces the neutron flux experienced by the screen with increasing thickness. Also, the screen itself is somewhat opaque to its own light, reducing the escape probability of the light with increasing thickness. These phenomena result in a diminishing proportionality between light output and thickness, and light yield can eventually decrease with thickness due to the neutron self-shielding.

Figure 7, Figure 8 and Figure 9 also illustrate the differences between the two methods of data analysis. While the linear assumption method is simpler and quicker to implement, it is less accurate than the pixel-by-pixel method. Generally speaking, the pixelwise correlation method curve fits theoretical behavior far better than the linear-thickness assumption method curve. This is especially true near the middle of the range of thicknesses because its assumptions fix the minimum and maximum thickness and assume the middle thicknesses change linearly, which is not necessarily true. Light yield is underestimated, particularly at lower thicknesses, for the linear-thickness assumption method because most defects on the screens surface, such as scratches, remove scintillator material. This along with possible fabrication-induced thickness variations may cause the thickness of the screen to be overestimated. The exaggerated behavior displayed by the linear-thickness assumption in Figure 9 at thicknesses between ~30 μm and ~100 μm is a result of the method’s inaccuracies for both light yield and detection efficiency measurements for thinner scintillator layers. 

## 6. Conclusions

Designing and testing multi-thickness or wedge-shaped neutron scintillator screens with this method significantly simplifies scintillator screen performance testing. Measurements of a single screen using this approach provides relative light yield and detection efficiency for a range of scintillator thicknesses from only a few radiographs and straightforward post-processing. Previous screen characterization efforts required multiple screens and measurements to provide the same information for discrete thicknesses.

This pixelwise correlation method could be extended to provide information about spatial resolution with an edge specimen placed in front of the test screen parallel with the thickness gradient to determine the modulation transfer function (MTF) as a function of thickness. Additionally, while this approach was developed and demonstrated for neutron scintillator screens, the same approach would be applicable for other types of scintillator screens as well, such as for fast neutrons, gamma-rays, and X-rays.

This approach could potentially accelerate screen development by manufacturers and allow screen users to efficiently determine the optimal screen parameters for their particular beam energy spectrum and imaging system. This work stands to increase the throughput of and reduce the cost of neutron scintillator screen testing which potentially benefits the entire neutron and X-ray imaging communities, as scintillator screens with improved performance can be developed and tested faster, leading to improvements in existing scintillator technology.
